# De Garengeot Hernia Diagnosed with Point-of-care Ultrasound

**DOI:** 10.5811/cpcem.2019.1.41170

**Published:** 2019-02-26

**Authors:** Lindsey Jennings, Brad Presley, Edward Jauch

**Affiliations:** Medical University of South Carolina, Department of Emergency Medicine, Charleston, South Carolina

## Abstract

De Garengeot hernias, defined as a femoral hernia containing the appendix, are rare. It is even uncommon to have an incarcerated de Garengeot hernia with associated acute appendicitis. We report a case of a 76-year-old female presenting to the emergency department for a right lower quadrant abdominal mass for four days. Physical exam was consistent with an incarcerated hernia. A point-of-care ultrasound revealed a non-compressible, blind-ended loop of bowel within the hernia sac, concerning for acute appendicitis within the mass. Computed tomography of the abdomen and pelvis confirmed the diagnosis of acute appendicitis within a femoral hernia.

## INTRODUCTION

Abdominal hernias and appendicitis are both common conditions evaluated in the emergency department (ED). However, encountering acute appendicitis within an incarcerated hernia is quite rare. Amyand hernias are inguinal hernias with the appendix contained within the hernia sac. De Garengeot hernias are femoral hernias with the appendix contained within the hernia sac. Both of these hernias can be associated with acute appendicitis. This case report highlights the diagnostic difficulties in identifying patients with appendicitis within a hernia and is one of the few reported cases of making this diagnosis with point-of-care ultrasound (POCUS).^1^,^2^

## CASE REPORT

A 76-year-old female with a past medical history of nephrolithiasis, thyroid disease, and osteopenia presented to the ED for right lower quadrant abdominal pain for the prior four days. She stated that she had experienced pain after lifting several flowerpots. On the day of presentation the patient noticed a mass in the right lower quadrant. She went to her primary care physician for evaluation; an attempt to reduce the hernia in the office was unsuccessful, so the patient was transferred to the ED for concern of an incarcerated hernia.

**Image 1:**
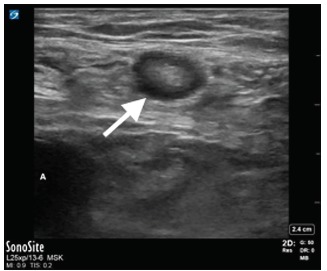
Appendix within the hernia sac. Arrow pointing to the appendix with a diameter of approximately one centimeter. Pressure is being applied with the ultrasound probe, demonstrating that the appendix is non-compressible, while also compressing the femoral vein so it cannot be visualized. The femoral artery can be seen in the lower left (A).

**Image 2:**
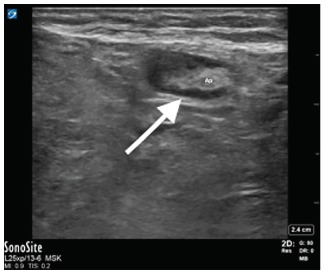
Oblique view of appendix on ultrasound. Arrow pointing to fluid around the appendix. *Ap*, appendix.

**Image 3:**
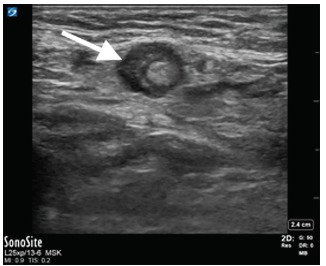
Point-of-care ultrasound image of appendix with hypoechoic rim (arrow).

In the ED, laboratory evaluation revealed an elevated white blood cell count at 13.02 millimeters (mm)^3^ (range 4.8–10.8 mm^3^), normal serum lactate of 0.57 millimoles per liter (mmol/L) (range 0.5–1.6 mmol/L), and a normal metabolic panel. A second attempt to reduce the mass after intravenous administration of hydromorphone was unsuccessful. Subsequent POCUS showed a blind-ended, non-compressible, dilated loop of bowel one centimeter in diameter within the right lower quadrant mass, concerning for acute appendicitis within the incarcerated hernia sac (Images 1, 2 and 3). Sonographic criteria for the diagnosis of acute appendicitis include a non-compressible, blind-ended loop of bowel that is greater than 6 mm in diameter without peristalsis.

**Image 4:**
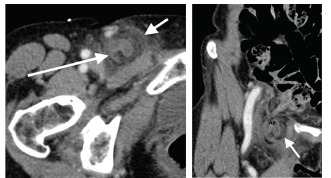
(Left) Axial view of the inflamed appendix within the hernia sac on computed tomography. Long arrow pointing to the appendix, short arrow pointing to the hernia sac. (Right) Coronal view of appendicitis within the hernia sac on computed tomography (arrow).

Given the concern for acute appendicitis within the hernia sac, no further attempts at reduction were made and surgery was consulted. A computed tomography (CT) of the abdomen and pelvis performed per surgery’s request confirmed the diagnosis of a right-sided femoral hernia containing an inflamed appendix, consistent with a de Garengeot hernia with acute appendicitis (Image 4). The patient went to the operating room for appendectomy and hernia repair.

## DISCUSSION

The presence of the appendix in a femoral hernia sac was first described in 1731 by the French surgeon de Garengeot, giving this type of hernia its eponym.^3^ This is not to be confused with an Amyand hernia, in which the appendix is contained within an inguinal hernia sac.^4^ De Garengeot hernias are rare, accounting for 0.5–3% of all femoral hernias.^5^,^6^ There are less than 100 known cases of de Garengeot hernias.^7^ It is even more uncommon to have acute appendicitis, which occurs in 0.08–0.13% of patients with de Garengeot hernias.^8^

**CPC-EM Capsule**

What do we already know about this clinical entity?

*De Garengeot hernias, femoral hernias containing the appendix, are rare and can be associated with acute appendicitis*.

What makes this presentation of disease reportable?

*The diagnosis was initially made with point-of-care ultrasound (POCUS). Diagnosis by POCUS is uncommon and has only been reported a few times in the literature*.

What is the major learning point?

*De Garengeot hernias can present indistinguishably from a femoral hernia. If the hernia is reducible, the diagnosis of appendicitis may be missed, leading to higher morbidity and mortality*.

How might this improve emergency medicine practice?

*This case demonstrates the utility of POCUS. Additionally, increased awareness of this disease may decrease the rate of delayed diagnosis*.

De Garengeot hernias are often difficult to diagnose and may clinically present indistinguishably from an irreducible femoral hernia.^9^ While de Garengeot hernias have been diagnosed on CT, and in a few other case reports with ultrasound, they are often missed on imaging and almost never diagnosed preoperatively.^10^–^13^ Due to the uncommon nature of this condition, the best technique of operative management remains unclear and the laparoscopic approach remains controversial.^3^,^7^,^14^,^15^

## CONCLUSION

Our case of a de Garengeot hernia containing an acutely inflamed appendix shows that this diagnosis can be made with ultrasound. While the sensitivity and specificity of ultrasound in making the diagnosis of appendicitis within a hernia is still unknown, providers may consider ultrasound and CT in their initial evaluations. Emergency physicians should consider both Amyand and de Garengeot hernias in the differential diagnosis for patients with inguinal or femoral hernias, as these diagnoses can be difficult to make. In the setting of appendicitis within a hernia, reduction of the hernia alone will not adequately treat the appendicitis, which can lead to significant complications. Additionally, the lack of symptoms of appendicitis can lead to a delayed diagnosis, resulting in a high frequency of perforated or gangrenous appendicitis, leading to increased morbidity and mortality.^3^,^16^ Maintaining a high level of suspicion may lead to earlier diagnosis and decreased complications. 14,17
